# Kisspeptin in the medial amygdala and sexual behavior in male rats

**DOI:** 10.1016/j.neulet.2016.05.042

**Published:** 2016-08-03

**Authors:** Rebecca Gresham, Shengyun Li, Daniel A Adekunbi, Minghan Hu, Xiao Feng Li, Kevin T O’Byrne

**Affiliations:** Division of Women’s Health, Faculty of Life Sciences and Medicine, King's College London, Guy’s Campus, SE1 1UL, UK

**Keywords:** Amygdala, Kisspeptin, Erection, Sexual behavior, Male, Rat

## Abstract

The medial amygdala (MeA) is crucial for sexual behavior; kisspeptin (Kiss1) also plays a role in sexual function. Kisspeptin receptor (Kiss1r) knockout mice display no sexual behavior. Recently Kiss1 and Kiss1r have been discovered in the posterodorsal subnucleus of the medial amygdala (MePD). We hypothesised that Kiss1 in the MePD may have an influence on male sexual behavior. To test this we bilaterally cannulated the MePD and infused kisspeptin-10 in male rats. This caused the rats to have multiple erections, an effect specific to Kiss1 receptor activation, because Kiss1r antagonism blocked the erectile response. When Kiss1 was infused into the lateral cerebroventricle, there were no observed erections. We also measured the plasma levels of LH when Kiss1 is infused into the MePD or lateral cerebroventricle; Kiss1 increased plasma LH to comparable levels when infused into both sites. We conclude that Kiss1 has a role in male sexual behavior, which is specific to the MePD.

## Highlights

• Kisspeptin in the posterodorsal medial amygdala evokes ex-copular erections in rats.
• The mechanism is kisspeptin receptor specific.
• Kisspeptin induced GnRH and LH release are not implicated.

## 1 Introduction

The medial amygdala (MeA) is a key brain region in sexual behavior. Lesions of the MeA suppress sexual behavior [1] and the extent of lesion correlates with the decrease in sexual behavior [2]. Chemosignals from estrous females stimulate non-contact erections (NCE) and MeA lesions also prevent these erections in male rats [3]. Chemosignals from estrous females activate the MeA in male hamsters, as seen by Fos expression [4]. Androgen receptors are found in the MeA, and testosterone or dihydrotestosterone are required for NCE stimulated by an estrous female [5] and blocking of androgen receptors in the MeA prevents NCE [6]. Administration of androgens directly into the posterodorsal subnucleus of the medial amygdala (MePD) does not cause spontaneous erection, without the presence of an estrous female [7] therefore, the MePD may be responsible for causing erections when activated by olfactory cues or chemosignals. In addition, impairment in erectile competence as a result of MeA lesion particularly for NCE is suggestive of the importance of the MeA on erectile function [3]. Ex-copula erections, those that occur without an olfactory stimulus, or NCE have been described as analogous to human psychogenic erections caused by erotica [8].

Kisspeptin (Kiss1) and its link to sexual function have been well studied in the anteroventral periventricular nucleus and the arcuate nucleus. Kiss1 and its receptor (Kiss1r) are crucial for normal reproductive function [9] and have recently been found within the MePD, but their role in sexual function in this area has not been explored [10], [11]. Kiss1r knockout male mice display no sexual behavior unless stimulated with testosterone [12], and even with testosterone the mice do not have a partner preference; they cannot decipher the chemosignals. Intracerebroventricular (ICV) administration of kisspeptin-10 (kp-10) in male rats dose-dependently increases plasma luteinising hormone (LH) and testosterone [13]. In men, intravenous infusion of kp-54 stimulates LH and testosterone secretion [14]. Long-term infusion of kp-54 can reactivate the photo-inhibited hypothalamic–pituitary–gonadal (HPG) axis by direct stimulation of gonadotropin-releasing hormone (GnRH) release in male hamsters [15].

We hypothesised that Kiss1 is involved in the MePD influence on male sexual behavior. To test this we infused kp-10, and its antagonist, directly into the MePD to examine the effect on male sexual behavior, specifically ex-copula erections, and to determine whether GnRH/LH is involved.‘

## 2 Materials and methods

### 2.1 Animals

Adult male (200–225 g) Sprague Dawley rats obtained from Charles River (Margate, UK) were individually housed under controlled conditions (12 h light, 12 h dark cycle, lights on at 0700 h: temperature 22 ± 2 °C) and supplied with food and water *ad libitum.* Before the animals underwent surgery they were given sexual experience, which involved housing the male with a normal cycling adult female for 1 week. All procedures were conducted in accordance with the United Kingdom Home Office Animals Scientific Procedures Act 1986.

### 2.2 Surgical procedures

Surgical procedures were performed under aseptic conditions, using general anesthesia—ketamine hydrochloride USP (100 mg/kg; Phizer Ltd., Kent, UK) and Rompun (10 mg/kg; Bayer AG, Leverkusen, Germany) via intraperitoneal injection. The animals (n = 30) were secured in a David Kopf stereotaxic frame (Tujunga, CA, USA) and fitted with bilateral guide cannulae (22 gauge; Plastics One, Roanoke, VA) directed toward the MePD for microinfusion of kp-10; the stereotaxic coordinates for implantation being 3.4 mm lateral, 3.14 mm posterior to bregma, and 8.6 mm below the surface of the dura according to the rat brain atlas of Paxinos and Watson [16]. A separate group of rats (n = 14) was implanted with unilateral guide cannula (22 gauge; Plastics One) directed at the left lateral cerebral ventricle for microinfusion of kp-10; the stereotaxic coordinates for implantation were 1.5 mm lateral, 0.6 mm posterior to bregma, and 4.0 mm below the surface of the dura according to the rat brain atlas of Paxinos and Watson [16]. These animals served as an additional control group for potential intra-MePD administered kp-10 leaking into the ventricular system to exert its effects on erectile function. The guide cannulae were secured using dental cement (Associated Dental Product, Swindon, UK) and fitted with dummy cannulae (Plastics One) to maintain patency. A stainless steel slotted screw (Instec Laboratories, Boulder, CO) was affixed to the surface of the skull posterior to the guide cannulae using dental cement. The rats were housed singly in a male only room and allowed 3 days of recovery before behavioral testing. Once behavioral testing was complete each rat was fitted with two indwelling cardiac catheters via the jugular veins, to facilitate serial blood sampling [17]. The catheters were exteriorized at the back of the head and enclosed within a 30-cm metal spring tether (Instec Laboratories) secured to the slotted screw. The distal end of the tether was attached to a fluid swivel (Instec Laboratories), which allowed the rat freedom to move around the enclosure. Experimentation commenced 3 days later. Correct cannula placement in the MePD was confirmed by microscopic inspection of 30 μm brain sections. Only data from animals with correct cannula placement were analyzed.

### 2.3 Ex-copula behavioural test

The ex-copula behavioral test was performed as described by Sach and colleagues [8]. The test arena was a Plexiglass cage (60 × 36 × 20 cm, with wood chip bedding, Techniplast, Italy). After 5 min habituation to the test arena, male rats were given bilateral intra-MePD injections of human kp-10 (10 pmol, 100 pmol or 1 nmol in 400 nl; Sigma-Aldrich, Poole, UK), or Kiss1r antagonist (Peptide-234; 5 nmol in 400 nl; Sigma-Aldrich) followed by kp-10 (1 nmol in 400 nl) 5 min later, Peptide-234 (5 nmol in 400 nl) or artificial cerebrospinal fluid (aCSF, 400 nl) as vehicle control over a 5 min period. Microinfusion was performed manually over 5 min for each drug using a 5 μl syringe (Hamilton, Bonaduz, Switzerland). For animals implanted with intracerebroventricular (ICV) cannulae, kp-10 (0.1, 1, or 5 nmol in 400 nl aCSF) was injected over 5 min as described above. The animals were observed for 30 min and the number of ex-copula erection was scored by the emergence of glans penis from the penile sheath and intensive penile grooming [8]. In preliminary studies, animals were observed for 2 h; however, no erections occurred after the first 30 min. A crossover design was used for treatments, with each animal being used on up to 3 occasions with a different dose on each occasion and a 1–3 day interval between treatments. All experiments started between 0900 and 1200 h.

### 2.4 Intra-MePD administration of kp-10 and kiss1r antagonist on LH secretion

On the morning of experimentation, intra-MePD injection cannulae were loaded with kp-10 as above; the distal end of the tubing, prefilled with aCSF was extended outside of the cage to allow remote microinfusion without disturbing the rat during the experiment. Microinfusion was performed manually over 5 min using a 5 μl syringe (Hamilton). One of the two cardiac catheters was then attached via the fluid swivel to a computer-controlled automated blood-sampling system, which allows for the intermittent withdrawal of small blood samples (25 μl) every 5 min for 6 h without disturbing the rats [18]. Once connected, the animals were left undisturbed for 1 h before sampling commenced between 1000 and 1100 h. After removal of each 25 μl blood sample, an equal volume of heparinised saline (50 U/ml normal saline; Wockhardt, Wrexham, UK) was automatically infused into the animal to maintain patency of the catheter and blood volume. After 2 h controlled blood sampling, kp-10, Peptide-234 or vehicle was infused intra-MePD over 5 min. Rats received a single dose of 100 pmol or 1 nmol kp-10 in 400 nl aCSF, bilaterally. Control rats received 400 nl aCSF. Blood sampling continued for further 4 h and samples were frozen at −20 °C until assayed for LH. For experiments involving ICV administration of kp-10, a single dose of 1 nmol in 400 nl aCSF was used, and blood sampling carried as described above.

### 2.5 LH assay

A double-antibody RIA supplied by the National Institute of Diabetes and Digestive and Kidney Diseases (Bethesda, MD) was used to determine LH concentrations in the 25 μl whole-blood samples. The sensitivity of the assay was 0.093 ng/ml. The intra-assay variation was 7.3%, and the inter-assay variation was 10%.

### 2.6 LH secretion analysis

The effect of pharmacological agents on overall LH secretion was calculated by comparing the area under the LH profile [area under the curve (AUC)] for the 2 h pre-treatment period with the 1st and 2nd 2-h post treatment periods using SigmaPlot version 13 (Systat Software, San Jose, CA). Statistical significance was tested using one-way ANOVA followed by Dunnett’s test. All data were shown as mean ± SEM. P < 0.05 was considered statistically significant.

## 3 Results

### 3.1 Behavioural response to kp-10 infusion into the MePD

**Fig. 1:**
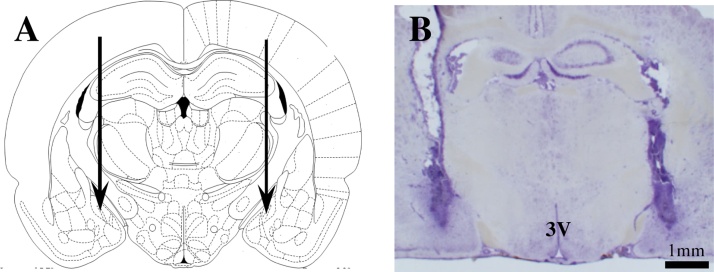
Schematic illustrations and photomicrograph of the cannulae target sites in the posterodorsal medial amygdala (MePD). A, Schematic illustration showing the target site for bilateral cannulation of the MePD at bregma (AP) −3.14 mm, mediolateral (ML) ± 3.4 mm and dorsoventral (DV) −8.6 mm according to the rat brain atlas of Paxinos and Watson [16]. Arrows point to the location of the cannulae tips. B, Photomicrograph of a coronal brain section in a representative animal implanted with bilateral cannulae in the MePD. 3 V, third ventricle.

Out of 30 rats, 22 with correct placement of bilateral cannulae in the MePD were verified by histological analysis with reference to the atlas of Paxinos and Watson [16] and included in the analysis. A representative example is shown in Fig. 1.

**Fig. 2:**
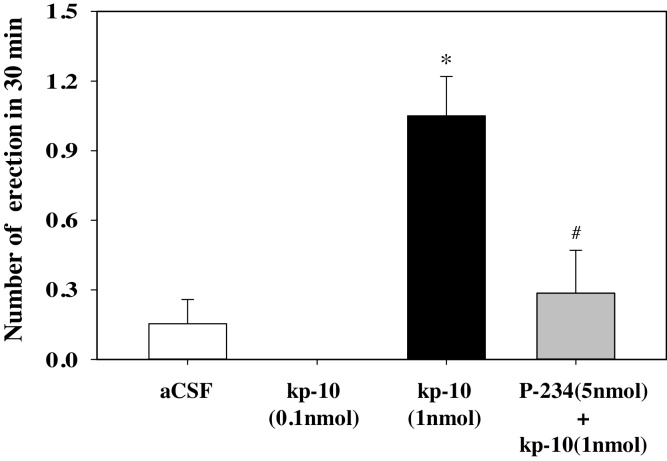
Effect of kisspeptin with or without kisspeptin antagonist injection into the posterodorsal medial amygdala (MePD) on the incidence of ex-copula erections. Bilateral administration of 0.1 nmol kisspeptin-10 (kp-10, n = 5) into the MePD had no effect on the incidence of ex-copula erections. However, 1 nmol kp-10 (n = 12) significantly increased the number of erections experienced, an effect blocked by kisspeptin receptor antagonism (P-234, 5 nmol; n = 6). Intra-MePD administration of artificial cerebrospinal fluid (aCSF) was withour effect (n = 12). *p < 0.05, compared with other treatment groups. ^#^p < 0.05, compared with 1 nmol kp-10 alone. Data presesented as mean and SEM.

An intra-MePD infusion of 1 nmol kp-10 caused a significant increase in the number of ex-copula erections compared with aCSF infusion (Fig. 2). All erections occurred between 8 and 27 min after infusion. The use of the Kiss1r antagonist, Peptide-234, prevented the erections (Fig. 2). These ex-copula erections were not observed with a 10 pmol (data not shown) or 100 pmol infusion of kp-10 (Fig. 2). There were no observed erections with 0.1, 1, or 5 nmol ICV infusions of kp-10 compared with aCSF controls (n = 5–8 per group) (data not shown). All animals implanted with ICV cannulae were correctly placed.

### 3.2 LH response to kp-10 infusion into the MePD

**Fig. 3:**
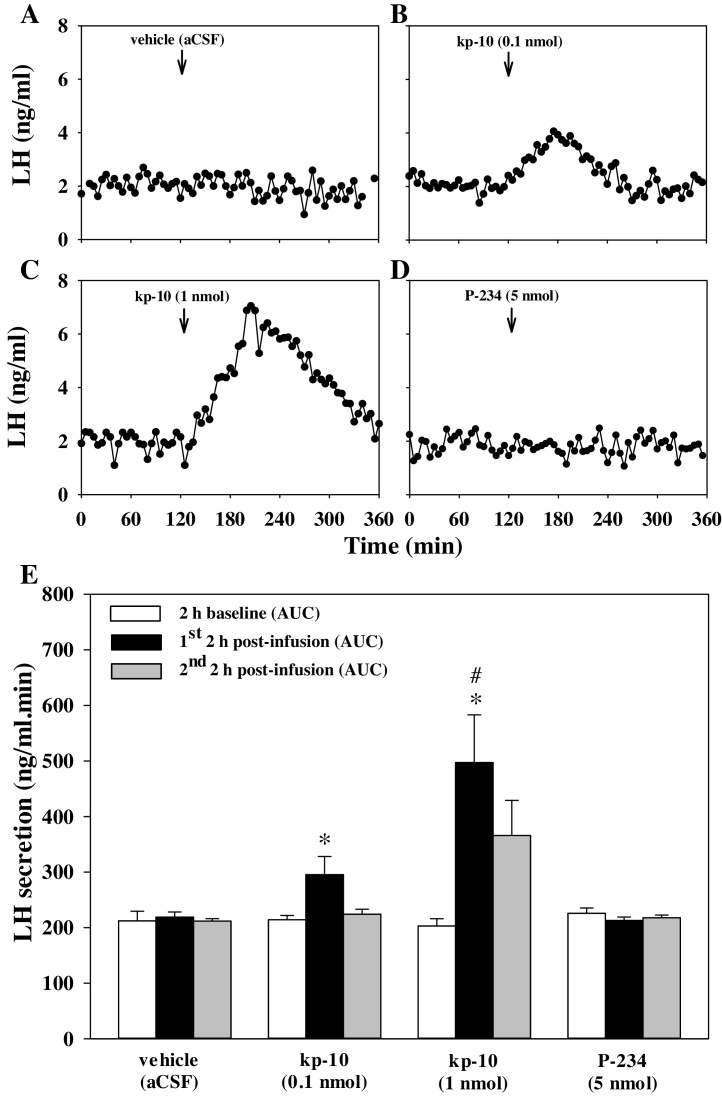
Effect of kisspeptin or kisspeptin antagonist injection into the posterodorsal medial amygdala (MePD) on LH secretion in male rats. Representative LH profiles demonstrating the effect of bilateral intra-MePD administration (↓) of vehicle (artificial cerebrospinal fluid; aCSF) (A), kisspeptin-10 (kp-10, 0.1 nmol) (B), kp-10 (1 nmol) (C), or kisspeptin receptor antagonist (P-234, 5 nmol) (D) on LH secretion. Direct administration of kp-10 into the amygdala dose-dependently increases LH secretion (B, C) shown by increased area under curve (AUC) of the LH profile (E). Kisspeptin antagonist had no effect on LH secretion (D, E). *p < 0.05, compared with vehicle. ^#^p < 0.05, compared with dose of 0.1 nmol kp-10 at same time point; n = 4–6 per group. Data presesented as mean and SEM.

Kp-10 dose-dependently increased plasma levels of LH in the first h after MePD infusion (Fig. 3), peaking at approximately 80 min post infusion for 1 nmol of kp-10. The Kiss1r antagonist had no effect on plasma LH (Fig. 3). An increase in plasma levels of LH was also seen 1 h post ICV infusion of 1 nmol kp-10, reaching comparable levels to those observed with intra-MePD of 1 nmol kp-10. ICV infusion of aCSF did not affect LH secretion (n = 5–8 per group) (data not shown).

## 4 Discussion

Intra-MePD infusion of 1 nmol kp-10 caused multiple ex-copula erections. The lower dose of 0.1 nmol kp-10 did not evoke an erectile response indicating that a dose threshold has to be reached to cause an erection. The erections were also blocked by Kiss1r antagonism indicating that the response is specific to Kiss1, and therefore may be a prerequisite for ex-copular erection to occur. Furthermore, evidence of the regional specificity of Kiss1 action is supported by the observation that ICV administration of kp-10 (0.1, 1, or 5–nmol) caused no sexual behavioral response, thus demonstrating that the MePD may be an obligatory structure for the pro-erectile effect of Kiss1.

Kiss1 infusion into the MePD caused a dose-dependent increase in plasma LH levels, as previously described for ovariectomized rats [19]. Despite ICV kp-10 resulting in a comparable rise in circulating levels of LH, only intra-MePD kp-10 evoked the erectile response. It can be concluded that the plasma LH rise, and consequently the Kiss1-induced GnRH secretion [20] is not related to the observed Kiss1-induced erections, despite GnRH previously been demonstrated to potentiate male sexual behavior [21]. Further to this, LH is still high 30 min post MePD infusion, but the erectile response at this point has ceased. Additionally, the lack of effect of intra-MePD administration of a Kiss1r antagonist on LH secretion, which is not dissimilar to the effect of ICV administration of peptide 234 in intact male rats and mice [22] supports the dissociation between changes in plasma LH and erectile function.

Interestingly, male rats with no previous sexual experience failed to respond to intra-MePD administration of kp-10 (data not shown), which suggests a significant context-dependent regulation of ex-copular erections involving amygdala Kiss1 signalling. The distinct mechanism by which Kiss1 signalling in the MePD facilitates the erectile response is yet unclear. The MePD is sexually dimorphic with a greater volume, synaptic input and dendritic spine density in male rats than in females, which is androgen dependent [23]. Additionally, *Kiss1* expression in the MePD is sexually dimorphic, with males having more *Kiss1*-expressing neurons, reflecting activational effects of the prevailing androgenic milieu [10]. Previous studies have shown that the exposure to female vaginal secretions can increase Fos expression in the MePD of male mandarin voles [24], and exposure to physically inaccessible females can selectively activate vasopressin neurons in the MePD of male rats [25]. Since MePD Kiss1 neurons receive putative innervation by vasopressinergic neurones [26], these observation suggest that MePD Kiss1 neurons may receive pheromonal cues controlling sexual behavior. Indeed, projections from the accessory olfactory bulb, which conveys pheromonal cues, densely innervates the MePD [27], and recently these projections were also shown to form close appositions to Kiss1 neurons in the MePD [26]. In addition to intrinsic vasopressin and Kiss1 interactions in the MePD, GABA signalling plays a role in sexual behavior [28], [29]. Intraperitoneal (IP) injection of a GABA_A_ agonist decreases sexual behavior; however, ex-copula erections were not specifically measured. IP injection of the GABA_B_ agonist, baclofen decreases sexual behavior almost totally [28]. Subcutaneous injection of baclofen inhibits ex-copula penile erections. However, mating can be achieved, so the inhibition can be overridden [29]. However, when GABA_B_ receptor is knocked out in mice, *Kiss1* mRNA increases in the MePD [30], suggesting that GABA_B_ regulates Kiss1 expression in the amygdala. This supports the hypothesis that a decrease in GABA_B_ receptor allows an increase in Kiss1 and an increase in sexual behavior. By infusing kp-10 into the MePD the GABA inhibition on Kiss1 has been potentially overridden causing erections.

In conclusion, Kiss1 infused into the MePD or ICV caused an increase in LH. However, only infusion into the MePD causes ex-copula erections, which can be analogised to a human erotica erection. We conclude that Kiss1 has a role in male sexual behavior, which is specific to the MePD, in addition to the regulatory function on gonadotropic hormone secretion.
